# Overexpression of Sirtuin 6 suppresses cellular senescence and NF-κB mediated inflammatory responses in osteoarthritis development

**DOI:** 10.1038/srep17602

**Published:** 2015-12-07

**Authors:** Yaosen Wu, Linwei Chen, Ye Wang, Wanli Li, Yan Lin, Dongsheng Yu, Liang Zhang, Fangcai Li, Zhijun Pan

**Affiliations:** 1 Department of orthopedics, Second affiliated hospital, Wenzhou medical university, Wenzhou, Zhejiang Province, China; 2 Department of orthopedics, Second affiliated hospital, Zhejiang University School of Medicine, Hangzhou, Zhejiang Province, China; 3 Center for Stem Cell and Tissue Engineering, Zhejiang University School of Medicine, Hangzhou, Zhejiang Province, China; 4 Department of orthopedics, affiliated hospital of Yangzhou University School of Medicine, Yangzhou, Jiangsu Province, China

## Abstract

The aim of our study was to evaluate if Sirt6, a NAD + dependent histone deacetylase, plays a protective role in cartilage degeneration by suppressing cellular senescence and inflammatory responses. The expression level of sirt6 in normal and OA human knee articular cartilage was compared by immunofluorescence and western blotting. The effect of sirt6 overexpression on replicative senescence of chondrocytes and NF-κB target genes expression was evaluated. Histological assessment of OA mice knee joint was carried out to assess the *in vivo* effects of sirt6 overexpression on mice chondrocytes. We found sirt6 level was significantly decreased in the articular chondrocytes of OA patients compare to normal human. SA-β-gal staining revealed that overexpression of sirt6 suppressed replicative senescence of chondrocytes. Meanwhile, the expression of NF-κB dependent genes were significantly attenuated by sirt6 overxpression. Safranin-O staining and OARSI score of knee joint cartilage in OA mice revealed that Lenti-Sirt6 intraarticular injection could protect mice chondrocytes from degeneration. These data strongly suggest that overexpression of Sirt6 can prevent OA development by reducing both the inflammatory response and chondrocytes senescence. Therefore, the development of specific activators of Sirt6 may have therapeutic potential for the treatment of OA.

Osteoarthritis (OA) is an age-related degenerative disease of the joint which is mainly characterized by the progressive degradation of cartilage with tissular inflammatory phase, along with joint pain and stiffness^1^,^2^,^3^. It affects millions of individuals across the world resulting in impaired quality of life and increased health costs. The roles of inflammation and aging in the pathophysiology of OA have been well established^4^,^5^,^6^. However, the underlying molecular mechanisms are not completely clarified yet. Therefore, studies on the mechanisms of aging and inflammation may also shed some light on understanding the pathogenesis or getting new therapeutic target of OA.

Sirt6 is a member of the sirtuins family (Sirt1-7) of histone deacetylases which has been implicated in aging, inflammatory and metabolic pathways^7^,^8^,^9^. The Sirt6 –/– mice exhibit postnatal growth retardation and have a greatly shortened lifespan and show degenerative and metabolic defects reminiscent of premature aging syndromes. Piao *et al.* identified that Sirt6 could regulate postnatal growth plate differentiation and proliferation^10^. Sirt6 deficiency inhibited chondrogenesis. Lee *et al.* revealed overexpression of Sirt6 could suppress inflammatory responses of synoviocytes induced by TNF-α^11^. Moreover, they demonstrated that Sirt6 inhibited bone destruction in collagen-induced arthritic mice by suppressing osteoclast differentiation^11^. However, to our knowledge, there is still no study identifying the roles of Sirt6 in cartilage or/and osteoarthritis.

In the present study, we hypothesize that Sirt6 has a significant relationship with OA development. Overexpression or activation of Sirt6 can prevent the progression of osteoarthritis. To test this hypothesis, we characterized the protective effects of Sirt6 in IL-1β-treated chondrocytes. The mechanistic pathway of Sirt6 involvement in chondrocytes denegeration was also investigated *in vitro*. The role of Sirt6 in OA development *in vivo* was studied in the model of mice OA knee joints.

## Material and Methods

### Reagents and antibodies

Dulbecco’s Modified Eagle Medium: Nutrient Mixture F-12 (DMEM/F-12) Media is obtained from Hyclone (Utah, USA). Penicillin, streptomycin and fetal bovine serum (FBS) were obtained from Gibco BRL (Grand Island, NY, USA). Recombinant human and mouse IL-1β were obtained from R&D Systems (Minneapolis, MN). The antibodies used in this study are as follow: anti-sirt6 from Abcam (Cambridge, MA); anti-NF-κB p65 and anti-IKb-α from Cell Signaling Technology (Danvers, MA); anti-actin from Sigma; anti-collogan II from Chemicon (Temecula, CA); anti-MMP-13 from Santa Cruz Biotechnology (Santa Cruz, CA); Alexa-Fluor-488- and Alexa-Fluor-545-tagged second antibodies were from Molecular Probes (Eugene, OR); secondary antibodies goat anti-rabbit IRDye 800CW and goat anti-mouse IRDye 680 were from LI-COR Biosciences (Lincoln, NE).

### Mice, human articular cartilage, chondrocyte culture

The C57 mice (Animal Center of Zhejiang University) were used in this study. Three-month and Twenty four-month old mice were used for immunohistochemistry analysis. Immature mice (10 days) were used for isolating knee articular chondrocyte as previously described method.

The normal human articular cartilage from 4 donors was obtained from femoral condyles and tibial plateaus at autopsy. OA human articular cartilage was obtained form 6 patients (OA grade III–IV) undergoing total knee arthroplasty. Cartilage slices were harvested from human joints. Human articular chondrocytes were incubated with 2 mg/mL of collagenase P in DMEM supplemented with 10% FBS and antibiotics at 37 °C overnight. After resuspension and filtration through a 0.7 μm filter, chondrocytes were cultured in a 24-well plate at a seeding density of 2 × 10^5^ cells/mL in DMEM/F12 supplemented with 10% FBS in an atmosphere of 5% CO2 at 37 °C^12^. Chondrocytes no later than first passage were used for follow-up experiments except in senescence-associated experiments. In these experiments, chondrocytes were passaged continuously to induce senescence.

### Senescence-associated b-galactosidase staining

Senescence-associated β-galactosidase (SA-β-gal) activity was detected with an SA-β-gal staining kit (Beyotime, Shanghai, China) following the manufacturer’s protocol. Senescent chondrocytes expressing SA-β-gal were stained blue.

### Immunofluorescence (IF)

Chondrocytes or slides of tissue sections were fixed in 4% formaldehyde. After washing three times in PBS, they were incubated in 10% FCS for 30 min to block nonspecific sites of antibody adsorption. Thereafter, the tissue sections or chondrocytes were incubated with appropriate primary antibodies overnight and secondary antibodies for 90 min. Images were captured on a Zeiss LSM510 Meta laser-scanning confocal microscope (Carl Zeiss, Thornwood, NY).

### Western blot analysis

The proteins lysed with sample buffer were loaded on 10% sodium dodecyl sulfate (SDS) polyacrylamide gel and blotted onto a PVDF membrane. Afterwards, the membrane was blocked in 5% BSA, and then incubated with the corresponding primary and secondary antibodies. After washing, the specific bands were analyzed using an Odyssey infrared imaging system (LI-COR Biosciences).

### Quantitative RT-PCR

Total RNA was isolated using Trizol (Invitrogen) according to the manufacturer’s instructions. cDNA was synthesized from 1 μg of RNA with One Step RT-PCR Kit (TaKaRa). Quantitative real-time PCR were performed using the iQTM SYBR Green supermix PCR kit with the iCycler apparatus system (Bio-Rad). The primer sequences were as follow: for MMP9, Forward 5′-AGATTCCAAACCTTTGAG-3′, Reverse 5′-GGCCTTGGAAGATGAATG-3′; for IL-6, Forward 5′-GAGAAAAGAGTTGTGCAATGGC-3′; Reverse 5′-ACTAGGTTTGCCGAGTAGACC -3′; for RANTES, Forward 5′-TGAAGATCTCCACAGCTGCAT-3′, Reverse 5′-CCTTCGAGTGACAAAGACGAC -3′; for GAPDH, Forward 5′-TCCCTCAAGATTGTCAGCAA-3′, Reverse 5′-GATCCACAACGGATACATT-3′. GAPDH was used as invariant housekeeping gene internal control.

### Lentivirus transfection

Lenti-SIRT6-Control, Lenti-SIRT6-WT and Lenti-SIRT6-H133Y lentiviral particles were produced by triple transfections of 293 T cells (Invitrogen, Carlsbad, USA) with the vectors of pLVX‐SIRT6-Control, pLVX‐SIRT6-WT, or pLVX‐SIRT6-H133Y, respectively, along with psPAX2 and pMD2.G.

Chondrocytes were transfected with lentivirus when cells reached 30–50% confluent at a multiplicity of infection of 200. More than 95% of the cells were still viable 12 h later and the culture medium was then changed. Three days later, all transfected cells were passaged for use in further experiments. The expression of SIRT6 was quantified by RT‐PCR and Western blot analyses.

### Animal OA model

Thirteen 4 month old mice were divided into 3 groups and anesthetized by intraperitoneal injection of chloral hydrate (250 mg/kg). Thereafter, OA+ Lenti-Sirt6 and OA+ Lenti-NC groups accepted transection of the medial collateral ligament and medial meniscectomy to induce surgical OA. Sham group accepted a sham operation using the same approach. Intra-articular injection of 10 μL Lentivirus was performed through a trans-patellar tendon approach at 0, 15, 30 and 45 days post OA surgery. Control groups accepted 10 μL Lenti-NC while experiment group was injected with Lenti-Sirt6. Mice were sacrificed at 8 weeks post-OA surgery from each group, the knee joints were dissected and processed for histological evaluation.

### Histological assessment

Knee joints were fixed in 4% paraformaldehyde, and decalcified in neutral 10% EDTA solution for 1 month at room temperature. Thereafter, the knees were embedded in paraffin blocks in the coronal position. Sections of 8 μm thickness were made for Safranin-O and Fast-Green staining^13^. The articular cartilage was assessed using the Osteoarthritis Research Society International (OARSI) histological scoring system^14^.

### Statistical analysis

Statistical analysis was performed using Stata 10.0 software (StataCorp LP, College Station, TX). Data are expressed as the mean ± standard deviation (SD). Statistical comparison was performed with a 2-tailed Student’s t test. Differences were considered significant when P < 0.05.

### Ethics Statement

This study was carried out in strict accordance with the approved guidelines for the Care and Use of Laboratory Animals of the National Institutes of Health. The protocol was approved by the Animal Care and Use Committee of the Medical Faculty of Zhejiang University. (Permit Number: XI104079). All surgery was performed under chloral hydrate anesthesia, and all efforts were made to minimize suffering. The human tissue collection was approved by the Ethics Committee of Second affiliated hospital, Zhejiang University School of Medicine. (Permit Number: 12377). Informed consents were obtained from all subjects.

## Results

### The expression of sirt6 in normal and OA human knee articular cartilage

**Figure 1:**
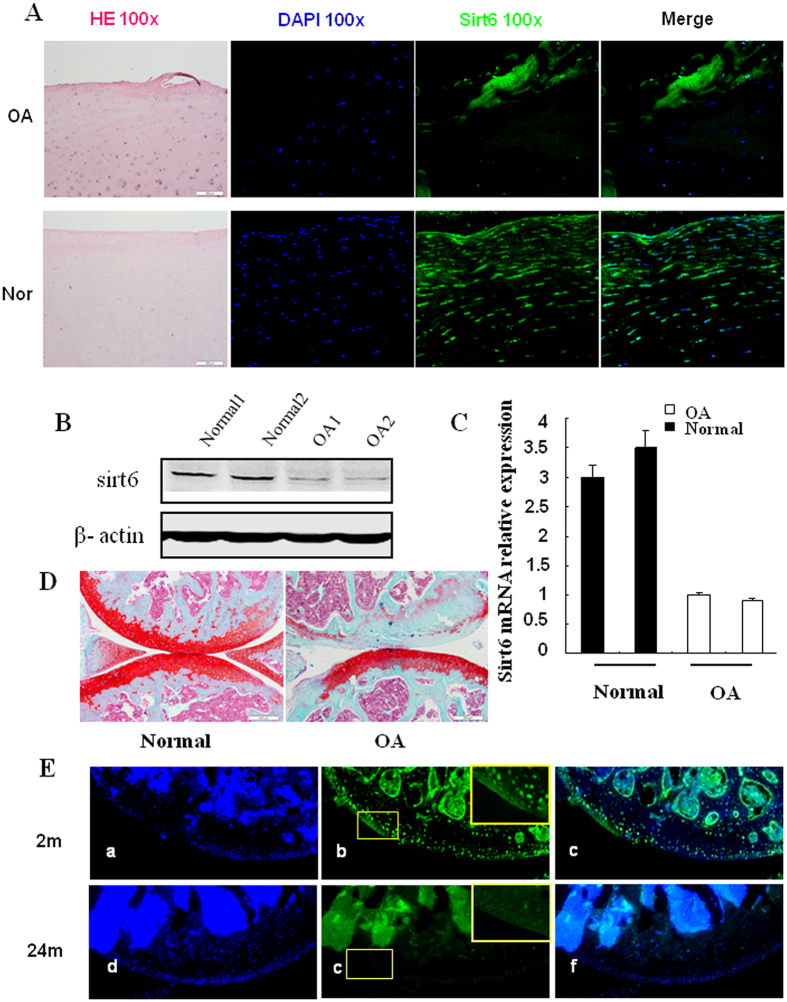
The expression of sirt6 in normal and OA knee articular cartilage. (**A**) Immunofluorescence analysis of human articular cartilage showed the sirt6 fluorescence intensities were significantly decreased in OA patients relative to normal. (**B**) The western blotting analysis revealed that the protein level of sirt6 was significantly decreased in the chondrocytes of OA patients. (**C**) The quantitative RT-PCR showed the expression of sirt6 was significantly lower in the chondrocytes of OA patients than in normal human. 1 & 2 indicated specimens from two people. (**D**) The thickness and integrity of the articular cartilage were decreased in OA cartilage compared with normal cartilage on safranin O stains. (**E**) The immunofluorescence stains of the mice cartilage revealed that the sirt6 fluorescence intensities were significantly decreased in aged mice relative to young mice.

To study the role of sirt6 in the development of OA, we first evaluated the levels of sirt6 in normal and OA human articular cartilage by immunofluorescence. As shown in Fig. 1A, the sirt6 fluorescence intensities are reduced in the knee articular cartilage of OA relative to normal. To confirm this result, primary human knee articular chondrocytes cultivated from normal and OA patient were studied by western blotting. Similar results were also observed, the protein and mRNA level of sirt6 was significantly decreased in the articular chondrocytes of OA patients (Fig. 1B,C).

### Aging-related reduction of sirt6 expression in mice articular cartilage

Osteoarthritis is a highly age-related process of joints. The histological analysis of the knee joints from C57 mice aged 3 and 24 months showed a reduction in the thickness of the articular cartilage on safranin O stains (Fig. 1D). The immunofluorescence analysis of knee joints from C57 mice aged 3 and 24 months showed a reduction in the fluorescence intensities of sirt6 in articular cartilage (Fig. 1E).

### The levels of sirt6 proteins decline with celluar senescence

**Figure 2:**
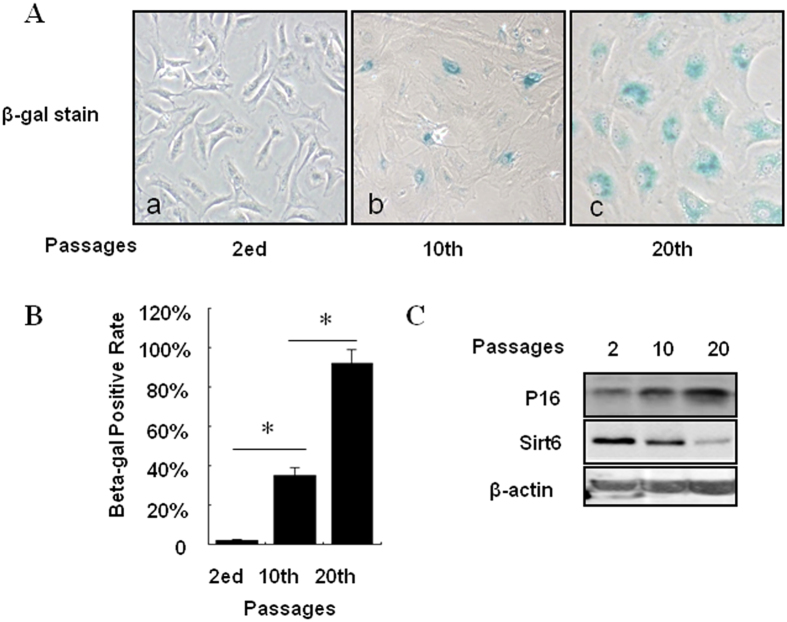
The levels of sirt6 proteins decline with celluar senescence. Celluar senescence was measured using SA-β-gal staining and p16 test. (**A**) SA-β-gal staining positive cells was found in chondrocytes at passage 10 and 20, but not presented in passage 2. (**B**) The percentage of SA-β-gal staining positive cells was dramatically increased in passage 20 compared to 10. (**C**) The expression levels of sirt6 and p16 were measured using western blotting at each passage.

To identify the levels of sirt6 associated with celluar senescence in chondrocytes, we examined sirt6 expression at the passage of 2, 10 and 20. Cell senescence was measured by SA-β-gal staining and p16, a specific marker of senescence cells. As shown in Fig. 2A, SA-β-gal staining positive cells was found in chondrocytes at passage 10 and 20, but not presented in passage 2. The percentage of SA-β-gal staining positive cells was dramatically increased in passage 20 compared to 10 (Fig. 2B). The expression levels of sirt6 and p16 were demonstrated by western blotting. Contrary to p16, sirt6 expression reduced significantly from passage 2 to 20 (Fig. 2C).

### Overexpression of sirt6 suppressed replicative senescence of chondrocytes

**Figure 3:**
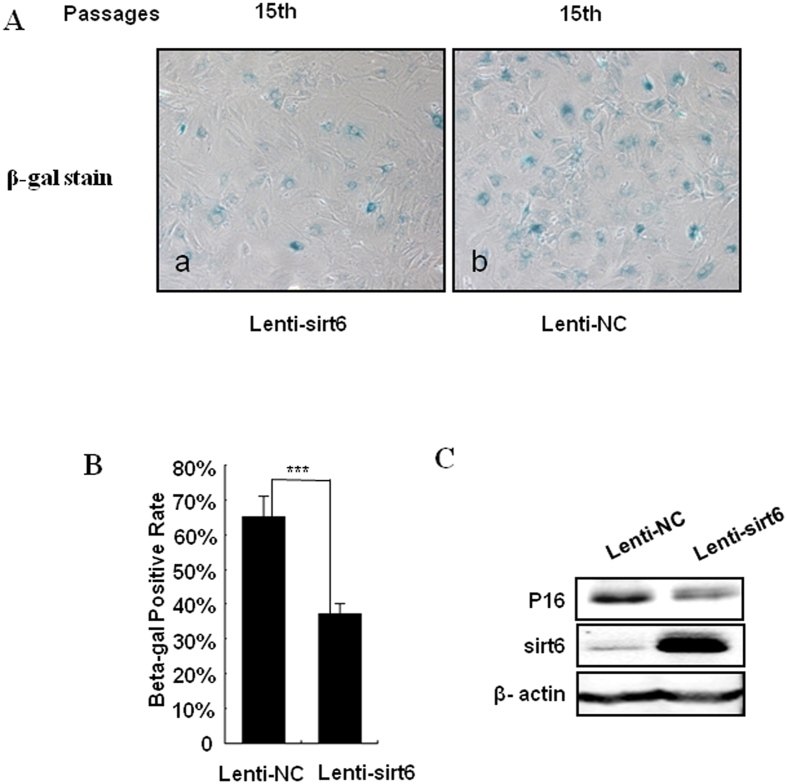
Overexpression of sirt6 suppressed replicative senescence of chondrocytes. (**A,B**) Successful overexpression of sirt6 decreased the percentage of β-gal staining positive cells. (**C**) Successful overexpression of sirt6 suppressed the expression levels of p16. *P < 0.05, **P < 0.01.

We have found that the levels of sirt6 reduced in the senescence chondrocytes. To clarify its regulatory role in replicative senescence of chondrocytes, successful overexpression of sirt6 was demonstrated by western blotting and SA-β-gal staining was used to mark senescence cells (Fig. 3A). Compared to sirt6 overexpression, we found that the percentage of SA-β-gal staining cells was significantly higher in non-overexpression group (Fig. 3B). Furthermore, p16 expression was demonstrated be decreased in sirt6 overexpression group (Fig. 3C). This result indicates that overexpression of sirt6 can suppress replicative senescence in chondrocytes.

### IL-1β reduces the expression of sirt6 in chondrocytes

**Figure 4:**
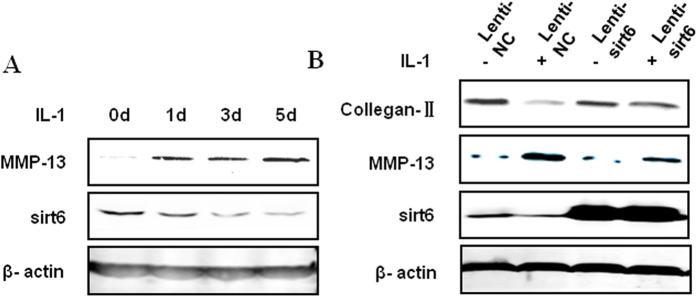
Effects of sirt6 overexpression on MMP-13 and collagen-II production in IL-1β induced chondrocytes. (**A**) Chondrocytes degeneration was induced after IL-1β treatment (10 ng/ml) for 0, 1, 3 and 5 days. The western blotting analysis revealed an increase in level of MMP-13 and a decrease in the level of sirt6 in a time-dependent manner. (**B**) MMP-13 level up-regulation after IL-1β treatment for 24 h was inhibited by the overexpression of sirt6. Meanwhile, colleagn II level was significantly decreased after IL-1β treatment and this effect was also significantly attenuated by sirt6 overexpression.

To elucidate whether the reduction of sirt6 expression was consistent with chondrocytes degeneration *in vitro*, we determined the expression of sirt6 after IL-1β treatment (10 ng/ml) for 0, 1, 3 and 5 days. As shown in Fig. 3, the western blotting analysis revealed that IL-1β treatment caused an increase in levels of MMP-13, meanwhile, reduced the expression of protein sirt6 in a time-dependent manner (Fig. 4A).

### Effects of sirt6 overexpression on MMP-13 and collagen-II production in IL-1β induced chondrocytes

To examine whether sirt6 plays a crucial role in the regulation of MMP-13 and type II collagen, we directly induced chondrocytes to express wild-type sirt6 using lentivirus (Lentivirus-sirt6). By using Western blotting, we first confirmed that the sirt6 protein was efficiently overexpressed. Then we examined the effect of sirt6 overexpression under the treatment with IL-1β (10 ng/ml) for 24 h. IL-1β stimulation significantly increased the MMP-13 level in chondrocytes. However, this up-regulation was inhibited by the overexpression of sirt6. Moreover, as show in Fig. 4, IL-1β decreased the expression of colleagn II and this effect was also significantly attenuated by sirt6 overexpression (Fig. 4B).

### Sirt6 overexpression has no effect on NF-kB-P65 nuclear translocation

**Figure 5:**
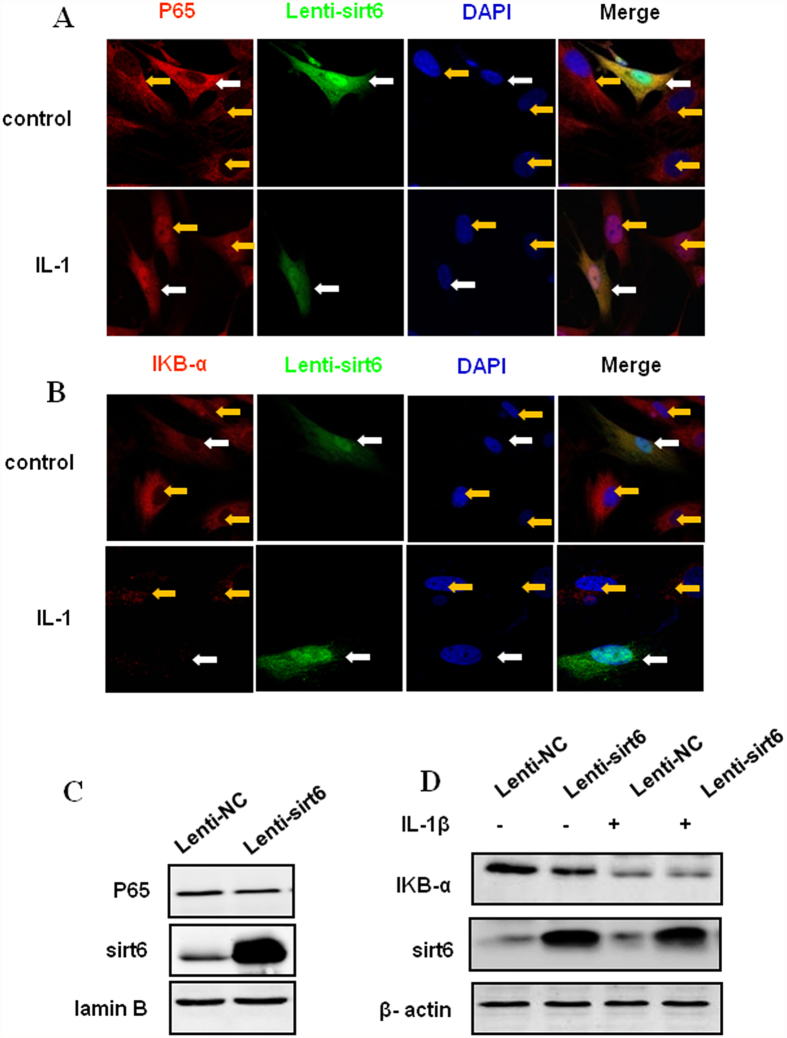
Sirt6 overexpression has no effect on NF-kB-P65 nuclear translocation. The mouse chondrocytes was firstly transfected with sirt6, thereafter cells was treated with IL-1β (10 ng/ml) for 24 h. (**A**) NF-kB-p65 translocated to the nuclear both in the chondrocytes with sirt6 transfection and non-transfection. (**B**) The degradation of Ik-Ba was noted in both kinds of chondrocytes after IL-1β treatment. (**C,D**) The result of western blotting analysis was consistent with that of immunofluorescence. Sirt-6 was co-transfected with GFP, therefore, GFP-positive cells represented the cells with Sirt6 over expression. White arrow and yellow arrows in Fig. A represent GFP positive cell and GFP negative cell respectively.

To address the mechanism by which sirt6 protects chondrocytes against IL-1β stimulation, the NF-kB pathway was examined since NF-kB is a well-known pathopoiesis factor for chondrocytes degeneration. Here the intracellular distribution of endogenous Ik-Ba and NF-kB-p65 after IL-1β treatment was studied by immunofluorescence and western blotting. Firstly the mouse chondrocytes was transfected with sirt6, thereafter cells was treated with IL-1β (10 ng/ml) for 24 h. As shown in Fig. 5A, NF-kB-p65 translocated to the nuclear both in the chondrocytes with sirt6 transfection and non-transfection. Meanwhile, the degradation of Ik-Ba was noted in both kinds of chondrocytes after IL-1β treatment (Fig. 5B). These results were further confirmed by our western blotting analysis (Fig. 5C,D).

### Effect of Sirt6 overexpression on NF-kB dependent gene expression

**Figure 6:**
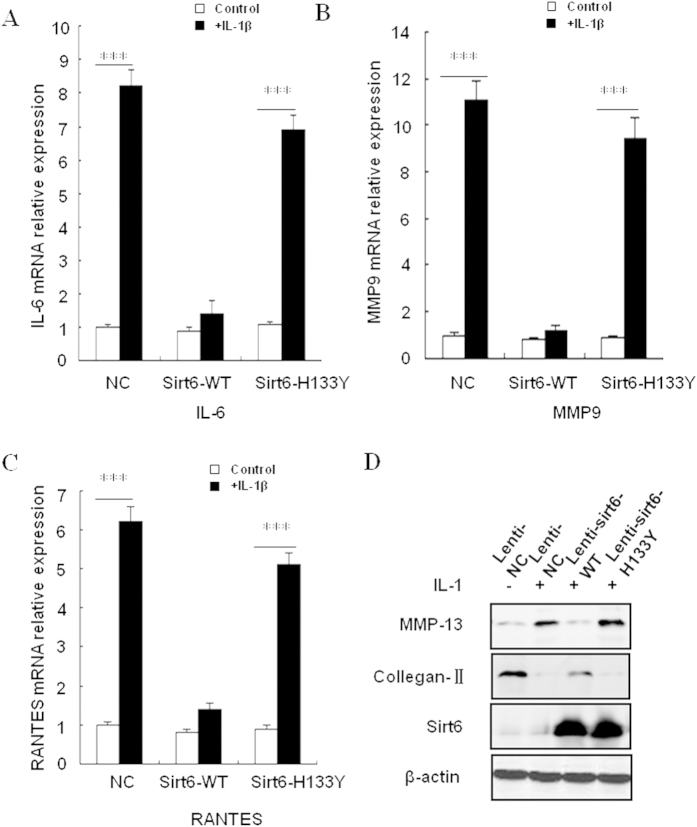
Effect of Sirt6 overexpression on NF-kB dependent gene expression. The mouse chondrocytes was firstly transfected with wild type sirt6 (sirt6-WT) and mutant type sirt6 (sirt6-H133Y), thereafter cells was treated with IL-1β (10 ng/ml) for 12 h. (**A–C**) The total expression of IL-6, MMP9, RANTES was measured using Quantitative RT-PCR. Compared with control group, the expression of NF-kB dependent genes were significantly attenuated by sirt6 overxpression. In contrast, sirt6-H133Y transfection didn’t inhibit NF-kB dependent genes. (**D**) Western blotting analysis revealed that MMP-13 expression and Collagen-II degradation were significantly attenuated by wild-type sirt6 overexpression. Meanwhile, this effect was not observed in sirt6 mutant group.

In the previous result we found that sirt6 overexpression has no effect on NF-kB-P65 nuclear translocation. In order to clarify the expression of NF-kB dependent gene, the mouse chondrocytes was firstly transfected with wild type sirt6 (sirt6-WT) and mutant type sirt6 (sirt6-H133Y). Catalytically inactive mutant H133Y was overexpressed to abolish the deacetylase activity of Sirt6. Then cells were treated with IL-1β (10 ng/ml) for 12 h. The total expression of IL-6, MMP9, RANTES was measured using Quantitative RT-PCR. Compared with control group, the expression of NF-kB dependent genes were significantly attenuated by sirt6 overxpression. In contrast, sirt6-H133Y transfection didn’t inhibit NF-kB dependent genes (Fig. 6A–C).

To further clarify the effect of Sirt6 overexpression on chondrocyte degeneration, we examined the expression of MMP-13 and Collagen-II in sirt6 transfected chondrocytes after IL-1β treatment. By using Western blotting, we confirmed that MMP-13 expression and Collagen-II degradation were significantly attenuated by wild-type sirt6 overexpression. Meanwhile, this effect was not observed in sirt6 mutant group (Fig. 6D).

### *In vivo* Effects of sirt6 overexpression on mice chondrocytes

**Figure 7:**
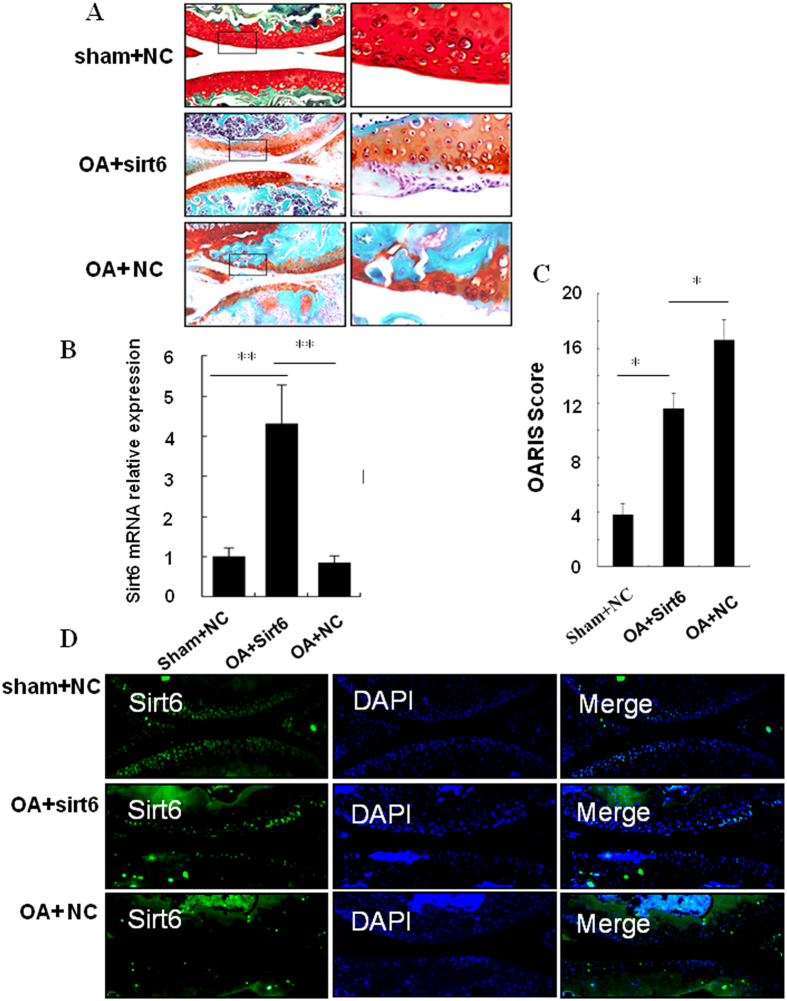
*In vivo* Effect of sirt6 overexpression on mice chondrocytes. (**A**) At 8 weeks after OA surgery, sham-operated group showed smooth cartilage surfaces and conserved SO staining in knee joint. OA+Lenti-NC group exhibited extensive cartilage missing and SO staining loss. Meanwhile, localized superficial cartilage loss was observed in the OA+Lenti-Sirt6 group. (**B**) RT-PCR result showed lentivirus-mediated Sirt6 overexpression was successful at 2 weeks after lenti-virus injection. (**C**) OARSI score was lowest in sham group and highest in the OA+Lenti-NC group. (**D**) The sirt6 fluorescence intensity in the OA+Lenti-Sirt6 group was significantly stronger than that in the OA+Lenti-NC group.

We investigated the potential of sirt6 Lentivirus to protect articular cartilage when injected into the knee joint of mice. At 8 weeks after surgery, sham-operated group showed smooth cartilage surfaces and conserved SO staining in knee joint. Chondrocytes arrangement was conserved as well, with one or two layers of tangentially arranged cells in the superficial zone and columns of round cells in the deep zones. By contrast, operated knees in the OA+ Lenti-NC group exhibited classical OA features as evidenced by extensive cartilage missing, SO staining loss and disorganized chondrocytes organization. In addition, we observed localized superficial cartilage loss, reduction in the thickness of cartilage and SO staining pale in the OA+ Lenti-Sirt6 group (Fig. 7A). To confirm lentivirus-mediated Sirt6 overexpression was successful, cartilage samples were collected at 2 weeks after virus injection. RT-PCR analysis showed Sirt-6 was overexpressed in chondrocytes in Lenti-sirt6 group (Fig. 7B).

OARSI score were 1.4 ± 0.2 in sham group, 7.2 ± 1.8 in the OA + Lenti-Sirt6 group and 17.5 ± 3.1 in the OA+ Lenti-NC group, respectively. The difference was significant in each group (Fig. 7C).

To verify the transfection effect of the Lenti-Sirt6 on chondrocytes, we observed the immunofluorescence of sirt6 in the cartilage. The sirt6 fluorescence intensity in the OA+ Lenti-Sirt6 group was significantly stronger than that in the OA+ Lenti-NC group (Fig. 7D).

## Discussion

Sirtuins are members of a family of evolutionarily conserved enzymes with NAD+-dependent deacylase activity which have been implicated in influencing a wide range of cellular processes including inflammation, apoptosis, aging, metabolism and stress resistance^15^,^16^,^17^,^18^,^19^,^20^. In Mammalian, sirtuins have seven isoforms (Sirt1-7). They have been shown beneficial effects against many degenerative or inflammatory diseases. Recent studies from different groups illustrated that Sirt1, Sirt2 and Sirt6 could suppress inflammatory responses in collagen-induced arthritis^21^. Moreover, there are mounting evidences that increasing of Sirt1 activity appeared to be a promising strategy for OA treatment^22^,^23^. However, it remains unknown whether Sirt6 participates in the development of osteoarthritis. In the present study, we identify that Sirt6 can effectively suppress cartilage degradation both *in vivo* and *vitro*. Our results strongly indicate Sirt6 as a considerably inhibitor of OA development.

Sirt6 could suppress cellular senescence in many tissues^9^,^24^. Minagawa *et al.* reported overexpression of Sirt6 efficiently inhibited epithelial cell senescence in idiopathic pulmonary fibrosis (IPF)^25^. Sharma *et al.* found that Sirt6 played important roles in the aging and reprogramming of human induced pluripotent stem cells (IPS)^26^. *In vivo*, increasing Sirt6 expression through genetic manipulation extends the lifespan of nematodes, flies and male mice^9^. In keeping with its role in aging, our study identifies that Sirt6 can suppress chondrocytes replicative senescence. Since senescence of chondrocytes with associated phenotypic changes contributes to the incidence and progression of OA^27^, it is logical to suppose that increasing of Sirt6 activity in chondrocytes can inhibit the development of OA.

Elevated level of proinflammatory cytokines such as IL-1β and TNF-α has been observed in OA cartilage and synovial fluid which can in turn increase the expression of matrix metalloproteinases (MMPs) and degrade the collagen and proteoglycans of cartilage^28^,^29^. They have been shown to mediate cartilage degradation in humans as well as in animal OA models. Suppressing the proinflammatory cytokines can inhibit cartilage degradation^30^,^31^. Lee *et al.* demonstrated that overexpression of Sirt6 could suppress inflammatory responses in collagen-induced arthritic mice^11^. However, they did not clarify the roles of Sirt6 in chondrocytes. In the present study, we identified that the expression of Sirt6 was reduced in OA human cartilage compared to normal. *In vitro* cellular study, IL-1β was used to mimic the pathophysiology of OA. As expected, overexpression of Sirt6 in chondrocytes by lentivirus could significantly decrease the levels of MMP13 and preserve the expression of type II collagen. Our results indicate that Sirt6 can suppress IL-1β-mediated osteoarthritic changes of chondrocytes. Moreover, we confirmed the role of Sirt6 in OA development *in vivo* using mice OA knee joints. Piao *et al.* demonstrated that Sirt6 deficiency inhibited postnatal growth plate differentiation and proliferation in immature mice^10^. The present study identified that overexpression of Sirt6 could effectively ameliorate cartilage degradation in mature mice both *in vivo* and *vitro*.

The NF-κB proteins are a family of ubiquitously expressed transcription factors that play an essential role in apoptosis, cell senescence, inflammation, and immunity^32^,^33^. It is well established that the NF-κB signaling played a central role in the pro-inflammatory stress-related responses of chondrocytes^34^,^35^,^36^. It is considered to be an attractive therapeutic target in OA. Kawahara *et al.* indicated that Sirt6 could suppress NF-κB mediated inflammatory responses through deacetylating H3K9 on the promoters of NF-κB target genes^37^. Therefore, we hypothesized the protective role of Sirt6 against osteoarthritis was achieved through suppressing NF-κB signaling. To verify this hypothesis, we determined the NF-κB-dependent transcriptional activity. Our study indicated that overexpression of wild-type Sirt6 could significantly repress NF-κB-dependent transcriptional activity, and prevent chondrocytes osteoarthritic changes induced by IL-1β. However, these effects were diminished when overexpressed the SIRT6-H133Y which abolished the deacetylase activity. Thus we identified that the protective role of sirt6 in cartilage degeneration, at least in part, was attributed to its inhibitory effect on NF-κB-dependent transcriptional activity, and the deacetylase activity of sirt6 is critical for its protective function in OA.

In conclusion, the study indicates that Sirt6 acts as a crucial mediator of OA development. Overexpression of Sirt6 can prevent chondrocytes replicative senescence and osteoarthritic changes induced by interleukin-1β. Moreover, our results reveal that the anti-degenerative effect of Sirt6 dependents on its function of suppressing nuclear factor-κB signaling. Developing specific activators of Sirt6 may be beneficial for the prevention or therapeutic treatment of osteoarthritis.

## Additional Information

**How to cite this article**: Wu, Y. *et al.* Overexpression of Sirtuin 6 suppresses cellular senescence and NF-κB mediated inflammatory responses in osteoarthritis development. *Sci. Rep.* **5**, 17602; doi: 10.1038/srep17602 (2015).
